# Overlapping Features of Caudal Regression Syndrome and VACTERL Complex in a Neonate

**Published:** 2010-08-14

**Authors:** Lubna Ijaz, Afzal Sheikh

**Affiliations:** Department of Pediatric Surgery, The Children's Hospital and the Institute of Child Health Lahore, Pakistan

**Dear Sir**

Caudal regression syndrome (CRS) is characterized by a group of heterogeneous anomalies involving the distal spinal cord and vertebral column, genitourinary system, hind gut and limbs. The malformation may range from minor anomalies of spine and spinal cord to the extreme, the sirenomelia. Various authors pointed out an overlap of spectrum of anomalies in CRS and VACTERL (vertebral, anorectal, cardiac, tracheo-esophageal, renal and limb anomalies) complex [1 , 2 , 3]. A case of a neonate is presented in whom the spectrum of congenital anomalies overlapped between CRS and VACTERL complex.

A 1-day-old male neonate weighing 2.8 kg presented to the emergency room of our institution with imperforate anus and absent left lower limb. The baby was a product of consanguineous marriage. The newborn was vitally stable and examination revealed imperforate anus, left lower amelia, sacral agenesis, megameatus intact prepuce, left sided undescended testis (UDT) and a small tag of tissue in place of the absent limb (Fig. 1).

**Figure:**
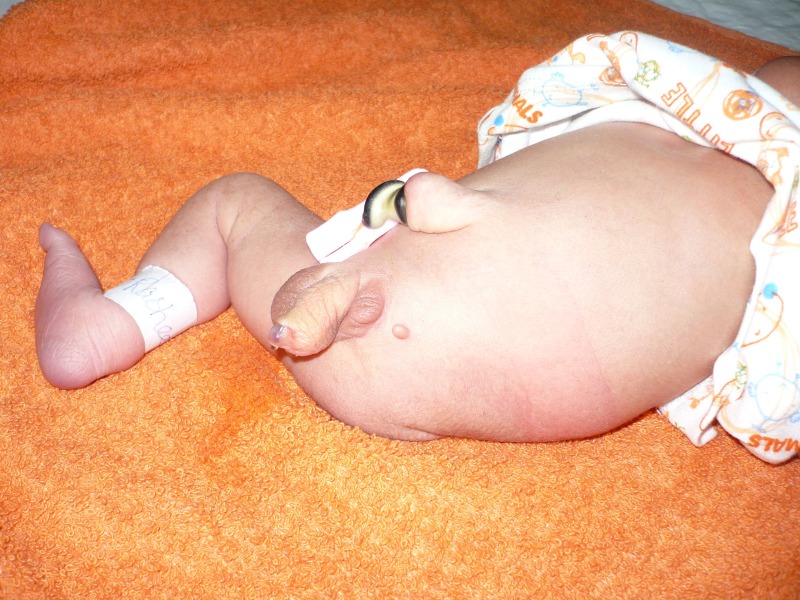
Figure 1: Photograph of the patient showing spectrum of anomalies including imperforate anus, left lower amelia, left UDT, megameatus and a skin tag in place of absent limb

Patient was admitted and routine neonatal care provided. Baby was subjected to various investigations. X-ray invertogram revealed high gas shadow in abdomen and sacral agenesis. Ultrasonography of the abdomen showed absent left kidney. Parents were counseled about the condition and a sigmoid loop colostomy performed for imperforate anus and discharged after two days.

CRS is a heterogeneous group of anomalies that may involve distal spine, spinal cord, genitourinary system, hind gut, and limbs; whereas, existence of VACTERL complex in a patient is considered where at least three of six anomalies of this complex are present [1 , 2 , 3]. 

Few authors have reviewed more than 150 patients of sirenomelia and depicted at least three out of six anomalies of VACTERL complex in more than 98% of the patients.

Shah et al1, in 2006, reported a case of sirenomelia associated with esophageal atresia and tracheo-esophageal fistula [1 , 4 , 5]. Our patient had four out of six anomalies of VACTERL complex and also fulfilled the criteria of caudal regression syndrome.

It therefore appears that caudal regression syndrome and VACTERL complex may be the same spectrum of anomalies that coexist in various combinations. As an overlap of the anomalies encountered, the use of the term caudal regression syndrome may be reserved for those cases having only caudal spinal and spinal cord anomalies, an event in isolation too rare, without multiple anomalies that usually overlap with the VACTERL complex. Moreover, it appears that both multi-anomaly-CRS and VACTERL anomalies may have similar etiological factors, a point that can be debated as more of such cases are reported.

## Footnotes

**Source of Support:** Nil

**Conflict of Interest:** None declared

## References

[1] ( 2006). Shah DS, Tomar G, Preetkiran. Sirenomelia. Indian J Radiol Imaging.

[2] ( 2007). Fayyaz A, Ilyas M, Iqbal O. Pre-natal diagnosis of caudal regression syndrome. J Coll Physicians Surg Pak.

[3] ( 2002). Taori KB, Mitra K, Ghonga NP, Gandhi RO, Mammen T, Sahu J. Sirenomelia sequence (mermaid): Report of three cases. Indian J Radiol Imaging.

[4] ( 1991). Duncan PA, Shapiro LR, Klein RM. Sacrococcygeal dysgenesis association. Am J Med Genet.

[5] ( 1987). Stocker JT, Heifetz SA. Sirenomelia: a morphological study of 33 cases and review of the literature. Perspect Pediatr Pathol.

